# Recent advances in understanding primary ovarian insufficiency

**DOI:** 10.12688/f1000research.26423.1

**Published:** 2020-09-07

**Authors:** Victoria Wesevich, Amanada N. Kellen, Lubna Pal

**Affiliations:** 1 Department of Obstetrics, Gynecology and Reproductive Sciences, Yale School of Medicine, New Haven, CT, USA; 2 Division of Reproductive Endocrinology and Infertility, Department of Obstetrics, Gynecology and Reproductive Sciences, Yale School of Medicine, New Haven, CT, USA

**Keywords:** Primary ovarian insufficiency, fertility, hypergonadotropic hypogonadism

## Abstract

Primary ovarian insufficiency (POI) is an uncommon yet devastating occurrence that results from a premature depletion of the ovarian pool of primordial follicles. Our understanding of both putative and plausible mechanisms underlying POI, previously considered to be largely “idiopathic”, has been furthered over the past several years, largely due to advances in the field of genetics and through expansion of translational models for experimental research. In this review, our goal is to familiarize the multidisciplinary readers of the F1000 platform with the strides made in the field of reproductive medicine that hold both preventative and therapeutic implications for those women who are at risk for or who have POI.

## Introduction

Despite the resounding progress that the field of reproductive endocrinology has witnessed over the past three decades, our understanding of the myriad of mechanisms causative to the entity of primary ovarian insufficiency (POI) remains lagging. Consequences of POI range from psychological devastation relating to the diagnosis to symptom burden, of which loss of fertility dominates the spectrum, to the long-term consequences of premature loss of ovarian function which include an increased lifetime risk for skeletal fragility and cardiovascular and neurocognitive disorders. Many gaps remain in our understanding of the processes regulating ovarian follicle quantity and quality, and what causes these processes to go awry, as occurs in POI. Until recently, the treatment modalities available to patients with POI were limited to the use of hormone therapy to mitigate symptom burden and minimize long-term risks of estrogen deprivation as well as the use of donor eggs as the only viable option for biological parenting. We recently reviewed the clinical presentation and diagnosis of POI ^1^. The purpose of the present review is to familiarize readers with the recent advances that have furthered our understanding of POI and which, in the foreseeable future, may even offer women with POI hope for biological parenting in every sense of the expression.

## Background

The prevalence of POI in the overall population is about 1%, although contributing to this background prevalence are the increasing rates of premenopausal cancer survivors with iatrogenic POI caused by gonadotoxic therapy ^2^. The diagnosis of POI is dependent on evidence of hypergonadotropic hypogonadism, namely elevated serum levels of the pituitary gonadotropin follicle-stimulating hormone (FSH) with low serum levels of estradiol (E2) in a patient who has irregular or absent menses and is younger than 40 years. When a POI diagnosis is suspected, serum levels of FSH and E2 should be measured twice, at least one month apart; persistently elevated FSH levels greater than 25 IU/L are consistent with POI ^3, 4^.

The clinical presentation of POI is highly variable. Changes in menstrual cyclicity (including prolonged or missed cycles, menstrual abnormalities, or amenorrhea), symptoms of hypoestrogenism (such as hot flushes, night sweats, vaginal dryness), and issues of subfertility or infertility are common presentations of POI. A finite spectrum of known causes of POI is summarized in Table 1. Notably, patients may manifest signs and symptoms (other than those of POI) that relate to the underlying etiology. For example, one or more of the phenotypic features of Turner syndrome (TS) may be evident, depending on the underlying genotype, in a POI patient who is missing one X chromosome in some (mosaic) or all cells. Similarly, symptoms of overt thyroid dysfunction or vitiligo, alopecia or hypoadrenalism may be evident in patients of POI if autoimmune underpinnings are deemed causative to ovarian follicle depletion ^5^. Once POI diagnosis is suspected, further workup is warranted to elucidate the etiology and identify covert comorbidities ( Table 2). The various causes of POI have different implications for long-term health; for example, a patient with a new diagnosis of TS is also at risk for existing cardiovascular structural abnormalities such as aortic coarctation, which if undiagnosed, may hold sinister health implications ^1^. Thus, recommended genetic testing includes karyotype and fragile X premutation testing; recommended screening for risk of autoimmune endocrinopathies includes testing for TSH and TPO and for 21-hydroxylase antibodies (if positive, identify the individual at an enhanced lifetime risk for autoimmune adrenal insufficiency).

**Table 1.** Known causes of primary ovarian insufficiency.

X chromosome disorders X chromosome deletions, inversions, duplications, balanced translocations Turner syndrome (XO and mosaics) Triple X syndrome (XXX) Fragile X (FMR1 gene premutation carrier) DIAPH2 translocation BMP15 variants PGRMC1 variants Genetic syndromes Ataxia telangiectasia Fanconi anemia Premature aging syndromes (Bloom and Werner) BPES Other single-gene variants See Table 3. Enzyme deficiencies Galactosemia (GALT) 17 alpha-hydroxylase Aromatase Autoimmune Polyglandular autoimmune syndrome types I and II Primary adrenal insufficiency Autoimmune thyroiditis Non-endocrine conditions (systemic lupus erythematosus, pernicious anemia, and myasthenia gravis) Infectious Viral (mumps, cytomegalovirus, and varicella) Bacterial (tuberculosis and shigella) Parasite (malaria) Toxins and injury Chemotherapy (for example, alkylating agent) Radiation Pelvic surgery (oophorectomy) Smoking

BMP15, bone morphogenetic protein 15; BPES, blepharophimosis, ptosis, and epicanthus inversus syndrome; CMV, cytomegalovirus; DIAPH2, diaphanous-related formin 2; FMR1, fragile X mental retardation gene 1; GALT, galactose-1-phosphate uridylytransferase; PGRMC1, progesterone receptor membrane component 1.

**Table 2.** Suggested workup for patients with primary ovarian insufficiency: elucidating the underlying etiology or identifying associated risks or both.

Tests	Relevance
**Serum hormones** Follicle-stimulating hormone, luteinizing hormone, estradiol Anti-Müllerian hormone Inhibin B Thyroid-stimulating hormone	Hypergonadotropic hypogonadism is a diagnostic criterion for primary ovarian insufficiency (POI) Marker of ovarian reserve-undetectable in POI Marker of ovarian reserve-undetectable in POI To assess thyroid function
**Genetic testing** Karyotype Fragility, mental retardation (FMR)	Aneuploidy/mosaicism/deletion/duplications Fragile X premutation carrier screening
**Autoimmunity screening** Thyroid autoantibodies Thyroid peroxidase antibody Thyroglobulin antibody 21-hydroxylase antibodies	Risk for thyroid dysfunction Risk for adrenal insufficiency
**Imaging studies** Ultrasound Ovarian antral follicle count Dual x-ray absorptiometry Bone mineral density	Ovarian reserve assessment Quantify lifetime risk for fracture

## Recent advances in the field of POI

### Diagnostic insights related to the genetic underpinnings of POI

**Figure 1.**
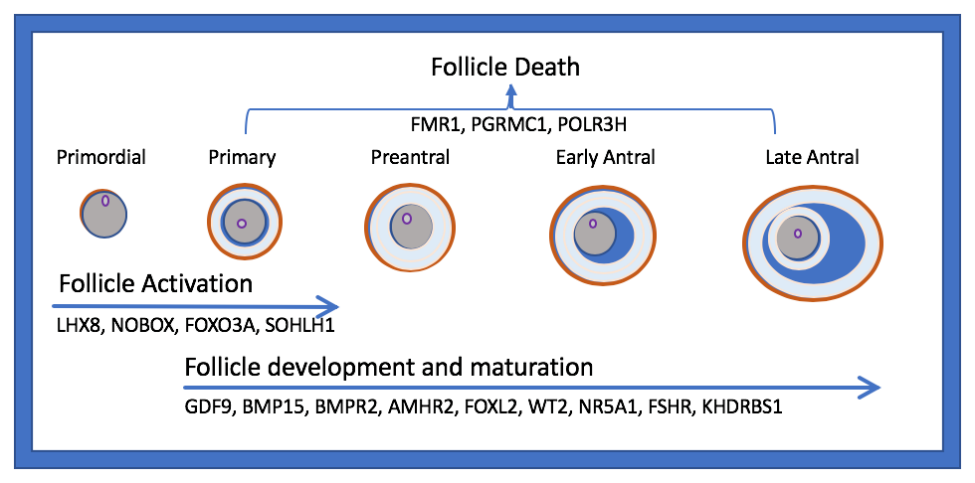
Selected genes involved in ovarian follicle activation, maturation, and death. *AMHR2*, anti-Müllerian hormone receptor 2; *BMP15*, bone morphogenic protein 15; *BMPR2*, bone morphogenetic protein receptor 2; *FMR1*, fragile X mental retardation; *FSHR*, follicle-stimulating hormone receptor; *FOXO3A*, forkhead box O3; *FOXL2*, forkhead box L2; *GDF9*, growth differentiation factor 9; *KHDRBS1*, heteronuclear ribonucleoprotein particle K homology domain RNA binding S1; *LHX8*, LIM homeobox 8; *NOBOX*, newborn ovary homeobox; *NR5A1*, nuclear receptor subfamily 5 group A member 1; *PGRMC1*, progesterone receptor membrane component 1; *POLR3H*, RNA polymerase III subunit H; *SOHLH1*, spermatogenesis and oogenesis specific basic helix–loop–helix 1.

Certain karyotypic abnormalities and single-gene mutations have long been recognized as causes of POI ( Table 3). In addition to the known contribution of X chromosome aneuploidies and mutations to the etiology of POI, increasing attention has focused on single genes known to regulate follicle development and maturation ( Figure 1), such as newborn ovary homeobox (NOBOX), bone morphogenetic protein 15 (BMP15), and growth differentiation factor 9 (GDF-9) ^6^. These genetic mutations (>60 identified thus far) demonstrate the complexity of the genetic architecture leading to this condition and offer targets for future genetic screening panels and possibly treatment modalities for women with idiopathic, sporadic POI. In efforts to detect novel POI genes, genotyping via genome-wide association studies (GWASs) and genome-wide sequencing through next-generation sequencing (NGS) have been used to identify genetic variations associated with POI. GWASs identify associations between genetic variation to a particular phenotype using many individuals with the disease or trait of interest. This evaluation is achieved by comparing common genetic variations known as single-nucleotide polymorphisms (SNPs), the most common genetic variations in the human genome in patients with POI versus unaffected controls. These SNPs may thus be used to identify genetic profiles that can be used to assess for causal relationships between phenotypes, many of which have been validated through *in vivo* or *in vitro* functional studies or both.

**Table 3.** Selected single genes associated with primary ovarian insufficiency.

Gene	Function
DNA damage repair genes	
*MCM8/9*	Required for homologous recombination-mediated repair of double-stranded breaks ^7^
*FANCA/M/C/G*	Required for S phase of growth cycle after exposure to DNA crosslinking agents ^8^
*RAD51*	Search for homology and DNA strand pairing; binds with BRCA1/2 ^9^
*ATM*	Cellular responses to genomic damage ^10^
*BRCA1/2*	Stimulates and maintains strand invasion within homologous recombination ^11– 13^
*PSMC3IP*	Meiotic recombination, coactivator of nuclear hormone receptor–dependent transcription ^14^
*STAG3*	Subunit of cohesion, required in meiosis for proper pairing and segregation of chromosomes ^15^
*NUP107*	Nucleoporin protein involved in transport between cytoplasm and nucleus, meiosis/mitosis progression ^16^
*SPIDR*	Recruits RAD51 complex in homologous recombination ^17^
*MSH4/5*	Complex that guides DDR toward crossover over non-crossover option ^17^
Ovarian function genes	
*BMP15*	Member of TGFB superfamily, regulates folliculogenesis ^18^
*GDF9*	Synergizes with BMP15; granulosa cell proliferation ^19^
*FIGLA*	E-box containing promoter ^15^
*FSHR*	Receptor to follicle-stimulating hormone, required for folliculogenesis ^20^
*POLR3H*	Regulates FOXO3A expression and subsequent primordial follicle activation ^21^
*NOTCH2*	Signal that regulates primordial follicle formation ^22^
*FOXL2*	Steroidogenesis, ovarian development, and maintenance ^23– 26^
*AHM/R*	Impair apoptosis repression ^27^

Of note, the above listed genes are not representative of a comprehensive list, as there are more than 60 which have been described. Rather, this list is intended to highlight genes have been most well-established and represent a broad spectrum of genetic functions contributing to POI pathogenesis. AMH, anti-Müllerian hormone; AMHR, anti-Müllerian hormone receptor; ATM, ataxia telangiectasia mutates; BMP15, bone morphogenetic protein 15; BRCA1/2, breast cancer gene 1/2; FANCA/M/C/G, Fanconi anemia complementation group A/M/C/G; FIGLA, folliculogenesis-specific basic helix–loop–helix transcription factor; FOXL2, forkhead box L2; FSHR, follicle-stimulating hormone receptor; GDF9, growth differentiation factor 9; MCM8/9, minichromosome maintenance 8/9; MSH4/5, MutS protein homolog 4/5; NOTCH2, neurogenic locus notch homolog protein 2; NUP107, nucleoporin 107; POLR3H, RNA polymerase III subunit H; PSM3IP, proteasome 26S subunit ATPase 3-interacting protein; RAD51, radiation-sensitive 51; SPIDR, scaffold protein involved in DNA repair; STAG3, stromal antigen 3.

Whereas GWAS uses traditional Sanger sequencing, which can analyze only 700 base pairs per reaction, NGS is a sequencing technique that enables the analysis of millions or billions of DNA strands in parallel. Whole exome sequencing (WES), a format of NGS, allows simultaneous analysis of base pairs across the entire exome and has been used in multigenerational POI families to identify single-nucleotide variants within genes known to be involved in ovarian function. From these studies, many potentially relevant variants from multiple family members can be rapidly investigated via gene panels using WES/NGS. Whereas GWAS is a more cost-effective method for common genetic variants, NGS allows a determination of the exact DNA sequence and can reveal information about all genetic variants, including those that are rare.

Once potential genetic candidates for POI have been identified, *in vitro* and *in vivo* techniques can be used for functional validation. One recent example of this is RNA polymerase III subunit H ( *POLR3H*), a gene that is highly expressed in developing spermatogonia, oocytes, and ovarian granulosa cells (which surround oocyte, which causes a POI phenotype) ^17^. In this study, two of eleven unrelated families with idiopathic POI were found to have a missense mutation c149.A>G via WES. In this setting, mouse models can be used for functional validation using clustered regularly interspaced short palindromic repeat (CRISPR)/CRISPR-associated system 9 (Cas9) technology to demonstrate functional effects of a particular mutation, which can readily investigate a particular gene’s dosage on phenotype, rather than complete loss of function via gene expression modulation. In that study, *Polr3h ^D50G^* knockout mice exhibited delayed puberty and decreased litter size and decreased expression of the ovarian transcription factor ** forkhead box O3 (FOXO3) and fewer antral and primary follicles ^17^. In a study of a family with four generations of women affected by POI, WGS identified a heterozygous nonsense mutation in an RNA polymerase II subunit, POLR2C ^21^; subsequent POLR2C knockdown in an embryonic carcinoma cell line resulted in decreased protein production and impaired cell proliferation. These data support a role for RNA polymerase II and III mutations as candidates in the etiology of POI. However, despite efforts to identify single genes causative for POI, it is likely that in many POI cases the disorder is more polygenic in nature. In a retrospective cohort study of 69 women affected by POI, WES identified 55 coding variants in 49 genes potentially related to POI; in 42% of the patients studied, at least two mutations in distinct genes were identified ^28^.

As the increasing use of GWAS and NGS has led to the identification of many genes potentially increasing an individual’s POI risk, the next step will be sifting through the data to better understand the synergy and specific relevance for these genes ^29^. As a clinical screening tool, genetic testing (beyond karyotype and FMR1 testing via WES/NGS) may help to associate a diagnosis of POI with a specific syndrome, cancer predisposition, or neurodegeneration. However, broad use of genetic testing (via WES/NGS) is controversial given the poorly understood phenotypic variation associated with each genetic mutation; if implemented, it is likely best performed in conjunction with a multidisciplinary team, including a genetic counselor and experts across multiple medical fields (for example, endocrinology, gynecology, and oncology). Furthermore, genetic testing can be more closely tailored via candidate gene sequencing if a monogenic syndromic condition is suspected on the basis of whether the patient is suspected to have a syndromic or non-syndromic phenotype ^30^. An ultimate clinical goal is the design of a targeted panel that can best describe a patient’s risk or even provide a diagnosis, allowing a more timely intervention.

### Genes involved in DNA damage repair

Mutations within the DNA damage repair (DDR) system represent intriguing candidate regulators of ovarian function ( Table 3). DNA double-strand break (DSB) repair and other DDR mechanisms are recognized for playing important roles in preserving the integrity of the follicle pool ^11, 31, 32^. Because the supply of available follicles is finite, the ability to detect and repair DNA damage is crucial for survival ^11, 31, 32^. Aging oocytes and follicles are less able to detect and repair DNA damage ^11, 12, 33, 34^. DNA damage is considered a “hallmark of aging”—a process that manifests during normal aging, accelerates the aging process when induced, and retards normal aging when blocked ^35^. DSB repair is critical in oocyte survival given the high frequency of DNA DSB which occurs during the meiosis portion of gametogenesis. Impaired DNA DSB repair, such as that observed in oocytes with deficiencies in RAD51 and ATM, blocks mouse oocyte maturation and results in oocyte death ^11^. Loss of oocyte DNA damage checkpoints also appears to be an important mechanism for the drastic increase in oocyte aneuploidy seen with aging ^36^.

It has been hypothesized that owing to the inheritance patterns of early menopause and POI within the same families, these two conditions are possibly manifestations of the same underlying genetic susceptibility with variable expressivities ^30^. Perhaps among the most well-known DSB repair genes are breast cancer type susceptibility protein 1 and 2 ( *BRCA1* and *BRCA2*). Mutations of both *BRCA1* and *BRCA2* have been associated with decreased ovarian reserve ^11^, failed follicular development and lower oocyte yield after ovarian stimulation in women undergoing assisted reproductive technology, and with an earlier age at natural menopause; conversely, in an experimental model, meiosis was restored with recovered levels of BRCA2 protein ^37^. Existing data are, however, limited by the heterogeneneity of the control groups, observational study designs, and small sample sizes ^32, 38^. Other DDR genetic mutations (for example, STAG3 [7q22.1] ^39^) have been shown to cause a premature depletion of the follicle pool, decreased primordial follicles, and increased recruitment and subsequent atresia/destruction of the growing follicles ^40^. Many other genes involved in the same DBS repair process have been discovered as potential gene candidates underlying POI. Other potentially pathogenic variants recently identified via WES of patients with POI include minichromosome maintenance 8/9 (MCM8 [20p12.3] and MCM9 [6q22.31]) ^7, 38, 41^. In particular, *MCM8*, a DNA DSB repair gene, has been implicated in the timing of menopause onset; a GWAS of about 70,000 women implicated MCM8 as well as HELB and SLC04A1 in the process of ovarian aging ^38^. Mutations in *PSMC3IP*, a gene regulating meiosis of germ cells and DSB repair, have been associated with the phenotype of primary amenorrhea and POI in a consanguineous family of four sisters with ovarian dysgenesis and a brother with azoospermia ^42^. Another interesting finding that overlaps two categories of POI—syndromic and non-syndromic (reviewed in 32)—is the recent finding of two missense variants of *FANCA* (the gene responsible for Fanconi anemia, or FA) in non-syndromic patients. FA genes are involved in DNA DSB repair ^14^ and are implicated in the category of syndromic POI; half of the patients with FA were reported to be infertile ^8^, and FA genes have been described as relevant variants of known causative gene mutations of gonadal dysregulation.

### Advances of therapeutic relevance for POI-associated infertility: toward a plausible “cure”?

A crucial question in ovarian biology is why some primordial follicles are maintained in dormancy for many years whereas others are activated for growth. Unlike in menopause, which occurs upon gradual depletion of the follicular pool via atresia or activation, residual primordial follicles unresponsive to standard gonadotropic signals remain in the ovaries. What is the mechanism by which adjacent follicles can be earmarked for such different fates? Current evidence suggests that whether an individual dormant follicle stays in the resting pool or initiates growth, transitioning out of a growth-arrested phase and developing into a mature peri-ovulatory follicle may depend on the balance of stimulatory and inhibitory factors at a particular point in time ^43^ but these factors remain poorly understood. A detailed understanding of the processes regulating timing of follicular activation would potentially allow the development of therapies designed to suppress this process and preserve ovarian function, which would be transformative for women with POI.

One exciting advance in POI is the exploration of stem cell therapy via residual follicle rescue ^44^. Live births have been achieved for POI patients with bone marrow transplant; this is thought to be due to replenishing factors necessary for an environment that facilitates growth within the ovary. One study injected bone marrow–derived stem cells (BMDSCs) and peripheral blood mononuclear cells into two groups of chemo-induced POI mice and immunodeficient mice with xenografted ovarian cortical fragments from poor-responder patients ^45^. The study showed many promising findings for both rodent and human ovarian tissue after BMDSC injection, including higher ovarian weight, higher ovulatory follicles, metaphase II oocytes, two-cell embryos, live births, estradiol secretion, and ovarian vascularization ^45^. Another recent study used an alternative stem cell source: endometrial mesenchymal stem cells (MSCs) injected into mice with chemo-induced POI resulted in higher circulating levels of anti-Mullerian hormone (AMH, a known marker of ovarian reserve), greater number of developing follicles, and higher ovulation and live birth rates ^46^. Furthermore, the therapeutic benefit of stem cells from other tissues, including adipose, umbilical cord blood, and amniotic epithelial cells, has been explored ^47– 50^. Recent research has begun to explore how stem cells specifically affect the ovarian microenvironment and restore ovarian function through the use of exosomes derived from stem cells in chemotherapy-induced POI models ^51, 52^. However, it is unclear at this point whether therapies aimed at prevention of POI in the oncofertility population will be broadly applicable to the larger POI population, including women with autoimmune and idiopathic POI and women in whom the onset of POI occurs early in life. 

Another approach that has been explored aims at increasing the concentration of local growth factors within the ovary utilizing intra-ovarian injection of platelet-rich plasma (PRP). Prepared from autologous plasma, intraovarian PRP has been shown to support the viability and growth of preantral follicles and to increase the number of retrieved oocytes ^53^. Growth factors that are thought to be delivered via PRP include platelet-derived growth factor (PDGF), epidermal growth factor (EGF), insulin-like growth factor (IGF), transforming growth factor b-I (TGFb-I), vascular endothelial growth factor (VEGF), hepatocyte growth factor (HGF), and basic fibroblast growth factor (bFGF) ^54^. This PRP-based tissue regenerative approach has been attempted across several injured human tissue types (tendons, muscles, and nerves) with the goal of improved healing ^55^ primarily through the promotion of neoangiogenesis. In a case report, the use of PRP with direct gonadotropin intraovarian injection resulted in successful conception in a POI patient who subsequently achieved a live birth ^56^. 

Ovarian tissue cryopreservation (OTC), in which a portion or the entire ovary is removed and frozen with the intention of autotransplantation, is a promising preventative option for fertility preservation in women planning initiation of chemotherapy or radiation ^57, 58^, especially for those with cancer diagnosed before puberty (and thus before controlled ovarian stimulation for oocyte cryopreservation can feasibly be achieved) (reviewed in 59– 63). Live birth rates have been calculated at about 25% across 80 patients following transplant of the thawed ovarian tissue at a later date; these pregnancies reflect a cumulative success and include both spontaneous conceptions and pregnancies resulting from *in vitro* fertilization ^64^. The majority of these patients underwent OTC for cancer-related POI risk (mostly, for breast cancer and leukemia diagnoses). However, it is unclear whether OTC studies largely consisting of patients with cancer can be extrapolated to patients with or at risk for POI relating to other etiologies, such as an abnormal genotype ^65^. A retrospective case-controlled study looking at ovarian biopsies in 15 patients with TS after OTC showed follicles in only nine of the ovaries (eight of these nine in Turner mosaic karyotype), along with markedly lower follicular fluid testosterone and estradiol concentrations ^65^, suggesting that a careful and patient-specific approach is essential when identifying TS patients who may benefit from OTC ^66^. 

**Figure 2.**
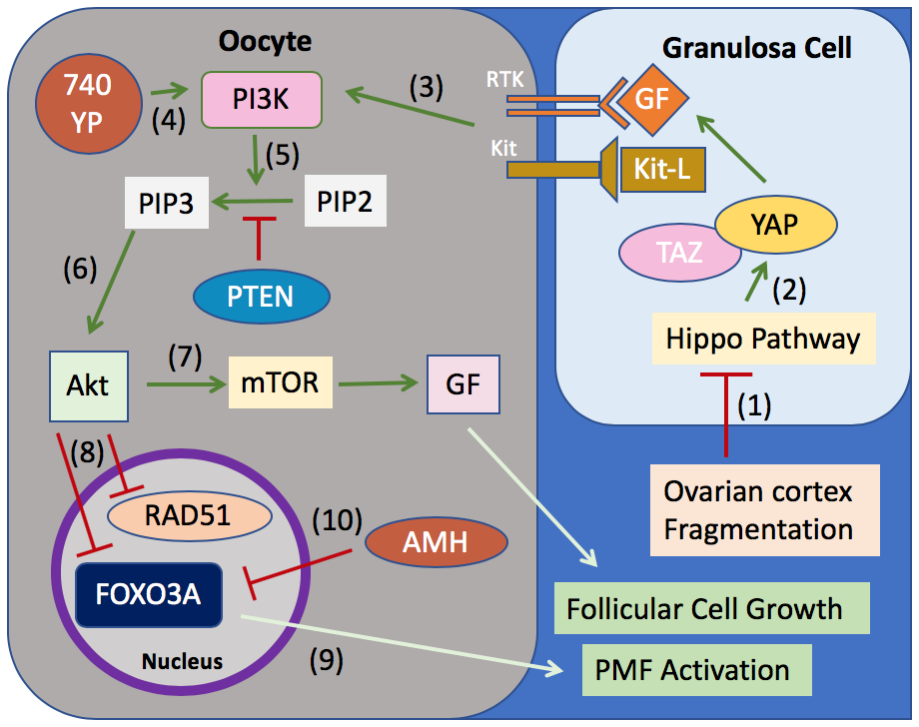
Selected pathways relevant for plausible salvaging of residual ovarian function in primary ovarian insufficiency. **(1)** Ovarian cortex fragmentation disrupts the Hippo signaling pathway leading to dephosphorylation of YAP and TAZ, which **(2)** stimulates transcription of growth factors (GFs), including GDF9 and BMP15 (transforming growth factor-beta family). **(3)** GF and Kit-ligand (Kit-L) as well as **(4)** 740YP administration increase PI3K activity, whereas PTEN serves to keep follicles dormant. **(5)** Activation of the PI3K complex activates PIP2 to PIP3, which **(6)** leads to increased Akt expression. **(7)** Phosphorylated Akt upregulates mTOR, leading to downstream GF transcription, and **(8)** inhibits activation of RAD51 and FOXO3A. **(9)** This prevents nuclear export of FOXO3A, decreasing primordial follicle activation. Similarly, **(10)** anti-Müllerian hormone (AMH) decreases activation of phosphorylation of FOXO3A. Green arrows represent activation steps, and red bar-headed lines represent inhibition. AKT, protein kinase B; FOXO3, forkhead box O3; mTOR, mammalian target of rapamycin; PI3K, phosphatidylinositol-3-kinase; PIP2, phosphatidylinositol-4,5-bisphosphate; PIP3, phosphatidylinositol-3,4,5-bisphosphate; PMF, primordial follicle; PTEN, phosphatase and tensin homolog deleted on chromosome 10; TAZ, transcriptional coactivator with PDZ-binding motif; YAP, Yes-associated protein.

Investigation into the mechanisms involved in the pathogenesis of POI are rapidly improving the potential for future POI treatments. A promising area of study as a therapeutic target involves the gene expression pathways regulating primordial follicle growth activation and recruitment with an eye toward oncofertility applications. Central regulators of this process include the c-Kit/Kit ligand signaling pathway, FOXO3, and members of the phosphatase and tensin homolog deleted on chromosome ten (PTEN) pathway, which modulate activity of protein kinase A (Akt) and mechanistic target of rapamycin (mTOR) and FOXO3 (reviewed in 43). In a study by Goldman *et al*., blockade of mTOR pathways with small-molecule inhibitors preserved ovarian reserve, primordial follicles, AMH levels, and fertility in a cyclophosphamide-treated mouse model ^67^. AMH, as a critical “gatekeeper” hormone that regulates follicular quantity by inhibiting recruitment and growth, is a particularly interesting molecule in the regulation of this follicular “clock” ^68– 73^. The use of exogenous AMH has been explored as an innovative new preventative strategy and treatment option for gonadotoxin-induced follicle loss ^74^. In two recent studies, co-treatment with exogenous delivery of AMH in chemotherapy-exposed mice resulted in an inhibition of primordial follicle growth activation, although the protective effect varied depending upon the agent used ^75, 76^. Because the PI3K signaling pathway is not activated by AMH, FOXO3 phosphorylation has been proposed as a causative mechanism for the protective effects of AMH ^75^ ( Figure 2).

*In vitro* activation has been proposed as a novel strategy for reactivating the dormant primordial follicle that still exist in POI ovaries. This concept is buoyed by recent advances in understanding of the role of the Hippo signaling pathway in activation of residual dormant follicles, and if substantiated, will hold transformative therapeutic relevance for patients diagnosed with POI ( Figure 2), (reviewed in 77). Disruption of the Hippo pathway caused by mechanical ovarian fragmentation has been used to activate resting follicles (although concerns about early activation and depletion of the follicular pool are also a consideration). In a study by Kawamura *et al*. using murine ovaries, *ex vivo* fragmentation of ovaries followed by reimplantation of fragmented tissue resulted in expression of key Hippo signaling genes and an increased percentage of late secondary and antral follicles, although an overall loss of follicles was observed after grafting ^78^. Moreover, when disrupted secondary mouse follicles were incubated with Akt-stimulating drugs (PTEN inhibitor and PI3K activator), similar increases in follicle counts were observed. Others have also demonstrated that primordial follicle activation (mediated via YAP1, the main downstream effector in the Hippo signaling pathway) is regulated in part by AKT ^79^. In humans, fragmentation of human ovarian tissue cubes followed by Akt stimulation also resulted in antral follicle growth when tissue strips were transplanted into immune-deficient mice. Subsequently, 27 patients with POI underwent ovarian tissue harvesting and fragmentation, and tissue was subjected to Akt treatment *in vitro* for 2 days, followed by auto transplantation beneath Fallopian tube serosa. Follicle growth was subsequently observed in eight patients and mature oocytes were retrieved from five patients; one woman achieved a live birth ^78^. In light of the understanding of the Hippo signaling pathway and its relevance for ovarian follicular development, it is plausible to consider that any benefits of intraovarian PRP injection on ovarian physiology may be secondary to a modulation of the Hippo pathway from physical disruption of the ovarian tissue (from injection), and not from intraovarian growth factors, as is hypothesized ^56^.

## Conclusions and Future directions

POI has historically been considered a poorly understood and catastrophic condition. Although the etiology of POI has largely been considered idiopathic, recent advances in the field of genetics have begun to unravel a complex network of molecular pathways that are critical to normal ovarian biology and that may be involved in the pathophysiology of this heterogeneous entity. Many potentially promising diagnostic and treatment modalities are already being explored. The overarching goal for the emerging tests and tools is to allow opportunities for an earlier diagnosis in those at risk of POI. Timely interventions may offer salvage of residual reproductive potential in the earlier stages of the POI processes. Elective freezing of oocytes and ovarian tissue are tried and tested interventions that allow for a preemptive approach to fertility preservation in those deemed at risk for POI, such as young girls and women with genetic predispositions (for example TS ^65^ and FMR1 premutation carriers ^81, 82^), pediatric and reproductive age cancer survivors ^80^, or women with autoimmune conditions ^83^.
